# Developmental Changes in the Inhibition of Glycinergic Synaptic Currents by Niflumic Acid in Hypoglossal Motoneurons

**DOI:** 10.3389/fnmol.2018.00416

**Published:** 2018-11-13

**Authors:** Elena Petukhova, Daria Ponomareva, Marat Mukhamedyarov, Galyna Maleeva, Piotr Bregestovski

**Affiliations:** ^1^Department of Normal Physiology, Kazan State Medical University, Kazan, Russia; ^2^Institute of Neurosciences, Kazan State Medical University, Kazan, Russia; ^3^INSERM, Institut de Neurosciences des Systèmes, Aix-Marseille University, Marseille, France

**Keywords:** niflumic acid, glycine receptors, anion-selective channels, hypoglossal motoneurons, patch-clamp recordings, brainstem slices

## Abstract

Mammalian brainstem hypoglossal motoneurones (HMs) receive powerful synaptic glycinergic inputs and are involved in a variety of motor functions, including respiration, chewing, sucking, swallowing, and phonation. During the early postnatal development, subunit composition of chloride-permeable glycine receptors (GlyRs) changes leading to a decrease of “fetal” alpha2 and elevation of “adult” alpha1 GlyR subunits. It has been recently demonstrated that niflumic acid (NFA), a member of the fenamate class of non-steroidal anti-inflammatory drugs, is an efficient subunits-specific blocker of GlyRs. At a heterologous expression of different GlyR subunits it has been shown that blocking potency of NFA is more than one order higher for alpha2 GlyRs than for receptors formed by alpha1 subunit. To reveal the action of NFA on the synaptic activity we analyzed here the effects of NFA on the glycinergic inhibitory post-synaptic currents in the HMs from mouse brainstem slices. In the whole-cell patch clamp configuration, the amplitude and the frequency of glycinergic synaptic currents from two age groups have been analyzed: “neonate” (P2–P4) and “juvenile” (P7–P12). Addition of NFA in the presence of antagonists of glutamate and GABA receptors caused a decrease in the mean amplitude and frequency of synaptic events. The degree of the inhibition induced by NFA decreased with the postnatal development, being higher on the motoneurons from “neonate” brainstem slices in comparison with the “juvenile” age group. Analysis of the pair-pulse facilitation suggests the post-synaptic origin of NFA action. These observations provide evidence on the developmental changes in the inhibition by NFA of glycinergic synaptic transmission, which reflects increase in the alpha1 and decrease in the alpha2 GlyR subunits expression in synapses to hypoglossal motoneurons during the early stages of postnatal life.

## Introduction

In the mammalian spinal cord and some other parts of CNS, inhibitory synaptic transmission is mediated by glycinergic synapses. IPSCs in this type of synapses originate from the presynaptic release of glycine that interacts with the post-synaptic GlyRs and induces opening of the anion-selective channels (Betz, 1991). GlyRs belong to the subfamily of Cys-loop receptors and consist of four alpha (a1–a4) and one beta subunits (Lynch, 2004; Betz and Laube, 2006). Functional receptors are represented by two forms: homometic GlyRs, consisting of alpha subunits, or heteromeric, formed by alpha and beta subunits. Beta subunit is required for the post-synaptic clustering of GlyRs (Kneussel and Betz, 2000).

Expression of the different receptor subtypes is developmentally regulated: “neonatal” alpha2 subunits are predominant at birth, but during the first weeks of postnatal development number of alpha2 GlyRs decreases, being partially substituted by “adult” alpha1 subunits (Akagi and Miledi, 1988; Becker et al., 1988; Malosio et al., 1991). This process is accompanied by the functional changes in the glycinergic neurotransmission – acceleration of the decay kinetics of IPSCs over the first weeks of postnatal period (Singer and Berger, 1999; Takahashi, 2005). This phenomenon is due to a shorter single-channel open time of the “adult” alpha1 receptors, comparing to the “neonatal” alpha2 GlyRs (Takahashi et al., 1992).

A recent study has demonstrated that NFA, a member of the fenamate class of non-steroidal anti-inflammatory drugs, is an efficient blocker of GlyRs with a subunit specificity of action (Maleeva et al., 2017). At heterologous expression of the different GlyR subunits it has been shown that a blocking potency of NFA was about one order higher for alpha2 GlyRs than for the receptors formed by alpha1 subunits. NFA activity was marked by the prominent voltage dependence. The compound had higher affinity to GlyRs at positive potentials, which was especially pronounced at alpha2 receptors.

To evaluate the effects of NFA on the function of glycinergic synapses, we performed an electrophysiological analysis of its action on the glycinergic IPSCs in the HM from brainstem slices of mice at the early stages of postnatal development. This brain region was selected since the motoneurons of hypoglossal nucleus (HN) generate powerful glycinergic post-synaptic currents (Singer and Berger, 1999; Donato and Nistri, 2000; Mukhtarov et al., 2005), providing a major inhibitory drive and controlling a variety of motor functions, including breathing, chewing, sucking, swallowing, and phonation (Lowe, 1980; Peever and Duffin, 2001).

The early work on the *in situ* hybridization analysis of the GlyR subunit mRNA distribution in HMs demonstrated the powerful changes during the first weeks of postnatal development (Singer et al., 1998). Predominant presence of alpha2 GlyR was detected at birth, while it decreased to a nearly background level at P18. In contrast, the expression of alpha1 GlyR subunit was low at birth and dramatically increased during the first 2 weeks of postnatal life. The expression of beta subunit was very high at all stages of postnatal development, while alpha3 GlyR was not expressed to any significant degree (Singer et al., 1998). This suggests that in the neonate mice, the HMs glycinergic synapses are predominantly composed of heteromeric alpha2/beta receptors, while the number of alpha1/beta GlyRs continuously increases during the first days of life.

For the examination of NFA action on the glycinergic synaptic transmission, we performed the electrophysiological recordings from HMs in brainstem slices of the mice at different stages of the early postnatal development addressing the following main questions:

1. whether NFA inhibitory action on the spontaneous glycinergic IPSCs changes during development;
2. how NFA inhibits glycinergic eIPSCs at the different membrane potentials;
3. 3.whether NFA modulates the neurotransmitter release from the presynaptic terminals.

We demonstrated that NFA causes an inhibition of spontaneous and evoked glycinergic IPSCs in HMs and its inhibitory activity decreases during the first weeks of postnatal life reflecting continuous developmental substitution of the “neonatal” GyRs subunits by the “adult” ones.

## Materials and Methods

### Animals

Experiments were performed on white laboratory ICR outbred mice of both genders of two age groups: neonatal (postnatal days P2–P4) and juvenile (P6–P10). Use of animals was carried out in accordance with the Guide for the Care and Use of Laboratory Animals (NIH Publication No. 85–23, revised 1996) and European Convention for the Protection of Vertebrate Animals used for Experimental and other Scientific Purposes (Council of Europe No. 123; 1985). All animal protocols and experimental procedures were approved by the Local Ethics Committee of Kazan State Medical University (N°742.13.11.84 and N°1045-72). Mice had free access to food and water and were kept under natural daylength fluctuations.

### Brainstem Slices Preparation

Mice were decapitated, the brainstems were removed and sliced into 250–400-μm-thick sections using a tissue slicer (model NVSLM1, World Precision Instruments). Sections were prepared in an ice-cold solution containing (in mM) 122 Choline chloride, 2.5 KCl, 1.25 NaH_2_PO_4_, 25 NaHCO_3_, 8 glucose, 0.5 CaCl_2_ and 7 MgCl_2_, saturated with 95% O_2_ and 5% CO_2_ (pH 7.3–7.4; 290–300 mOsm). Then slices were incubated for 1 h at a room temperature in a chamber filled with an oxygenated aCSF containing (in mM) 126 NaCl, 3.5 KCl, 2 CaCl_2_, 1.3 MgCl_2_, 1.2 NaHPO_4_, 10 glucose, 23.8 NaHCO_3_ (pH 7.3–7.4, 290–300 mOsm).

### Electrophysiological Recordings

For the recordings, the brainstem slices were placed in a chamber perfused with an oxygenated aCSF, containing CNQX, 10 μM and bicuculline 20 μM. The rate of perfusion was 25 ml/min. Recordings were performed at 30–31°C with the speed of perfusion 25 ml/min. The slices were visualized through a x60 water-immersion objective using an upright microscope Olympus BX51WI equipped with the Olympus DP72 CCD-camera. Hypoglossal motoneurons were identified by their localization within the HN (n. XII) and by their size (25–40 μm) and shape with a dendritic arborisation. Also after obtaining a whole-cell at the beginning of the each recording we determined the characteristic pattern of the action potential generation to a step depolarization in the current-clamp mode. Patch electrodes (resistance 4–5 MΩ) were filled with the intracellular solution containing (in mM): 110 K-gluconate, 30 KCl, 4 MgATP, 10 Phosphocreatine, 0.3 GTP, 10 HEPES, 5 EGTA (pH 7.3; 290 mOsm).

Membrane currents were recorded at 3–10 kHz using an EPC-10 patch clamp amplifier (HEKA Elektronik, Germany) at the whole-cell configuration of patch-clamp technique (Hamill et al., 1981). The holding potentials (V_hold_), in different experiments were -70 mV and/or +30 mV. For the induction of eIPSCs, the DS3 Constant Current Isolated Stimulator (Digitimer, England) was used and a bipolar stimulating electrode was placed on dorsolateral part of the HN. Current pulses 10–100 μA in amplitude and 100 μs in duration were applied. To avoid the direct stimulation of the recording MNs, a stimulating electrode was positioned at a distance >200 μm from the neuron of interest. To block the glutamatergic and GABAergic synaptic events, specific antagonists (10 μM CNQX and 20 μM bicuculline) were routinely added to aCSF.

Experiments on the analysis of NFA action on the spontaneous glycinergic IPSCs were usually performed using the following protocol: 5 min registration in the control conditions; 10 min – in the presence of 100 μM NFA; 10–15 min of the washing. For the analysis of eIPSCs the recordings were performed using the following scheme: 5 min in the control conditions, 3–5 min under the action of NFA and 10 min of the washing.

### Drugs

Niflumic acid (Sigma-Aldrich) was used at 100 μM concentrations freshly prepared from 100 mM stock dissolved in dimethyl sulfoxide (DMSO); bicuculline (Tocris), a GABAA-receptors antagonist; 6-cyano-7-nitroquinoxaline-2,3-dione (CNQX) disodium salt (Tocris), an AMPA glutamate receptors antagonist; strychnine (Sigma-Aldrich), an antagonist of GlyRs were freshly prepared from the 1000x stocks and applied at 20, 10, and 2 μM concentration, respectively. All stocks were kept at -20°C.

### Data Analysis and Statistics

Traces with spontaneous activity were analyzed with Igor Pro 6.02 (WaveMetrics, United States). Analysis of the amplitude and frequency of IPSCs was made with the Peak Analysis function from the Igor Pro^TM^ Tool Collection “Patcher’s Power Tools” (Max-Planck-Institut für Biophysikalische Chemie, Germany). Origin 9.0 software was used to perform a statistical analysis of the data and to plot the graphs. Decay times of the original evoked IPSCs (eIPSCs) were analyzed using Clampfit (Molecular Devices, United States) by determining the time in which the peak amplitude is reduced by e-times. At the amplitude analysis of eIPSCs, about 10 pair-pulse events were usually accumulated for the measurements.

Data are represented as means ± SEM. Statistical significance was determined using One-way ANOVA test with Bonferroni correction. Distributions of the peak amplitude were compared by Kolmogorov–Smirnov test. Differences were considered significant at *P* < 0.05.

## Results

### Synaptic Glycinergic Currents in the Hypoglossal Motoneurons

**FIGURE 1:**
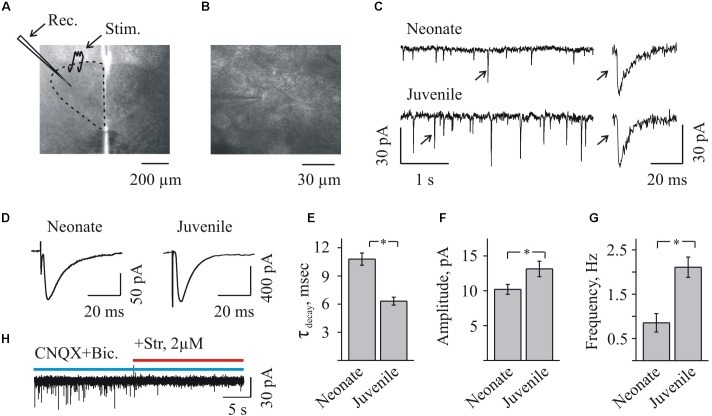
Whole-cell recordings of glycinergic synaptic currents from HMs in brainstem slices from mice of different ages. **(A)** Photo of brainstem slice showing the area of hypoglossal nucleus (HN) and the location of recording (Rec.) and stimulating (Stim.) electrodes. Age P3. **(B)** Microphotograph of HN ar higher amplification showing the recording electrode on the motoneuron. Age P8. **(C)** Traces of whole-cell recordings illustrating spontaneous glycinergic IPSCs, recorded from neonatal (P3, top trace) and juvenile (P10, bottom trace) mice. On the right single events are shown at higher resolution. Here and in the other figs all recordings are in the presence of CNQX 20 μM and bicuculline 20 μM. V_hold_ = –70 mV. **(D)** Examples of glycinergic eIPSCs recorded from motoneurons in neonatal (P3) and juvenile (P10) mice brainstem slices. Averaged traces of 10 individual eIPSCs induced by presynaptic stimulation are presented. Notice faster decay kinetics of IPSCs in the juvenile motoneuron. V_hold_ = –70 mV. **(E)** Summary of the average decay time constants of glycinergic eIPSCs in neonatal (*n* = 8) and juvenile (*n* = 10) groups. Values are mean ± SEM. **(F,G)** Summary of the mean amplitude and frequency of spontaneous glycinergic IPSCs in neonatal and juvenile groups (*n* = 6). Values are mean ± SEM. ^∗^ Significant difference (*P* < 0.05) (one-way ANOVA test with Bonferroni correction). **(H)** Trace illustrating complete inhibition of spontaneous IPSCs, at addition of strychnine to aCSF containing CNQX and bicuculline. V_hold_ = –70 mV. Age P12.

Using the whole-cell configuration of the patch-clamp recordings, the effect of NFA on synaptic glycinergic currents was studied on the hypoglossal motoneurons (MNs) in slices from the brains of mice of different age (Figures 1A,B). For the convenience of the analysis and data presentation we conditionally named two age groups as the “neonate” (P2–P4), and the “juvenile” (P7–P12), slightly changing the previous classification (Singer and Berger, 1999). To accelerate a data recording in some cases we used the simultaneous measurements from two motoneurons (Figure 1B). Effects of NFA were studied using two types of the recording protocols: (i) analysis of the amplitude and frequency of the spontaneous synaptic glycinergic currents (sIPSCs) (Figure 1C) and (ii) analysis of the synaptic currents induced by a presynaptic stimulation (Figure 1D).

**FIGURE 2:**
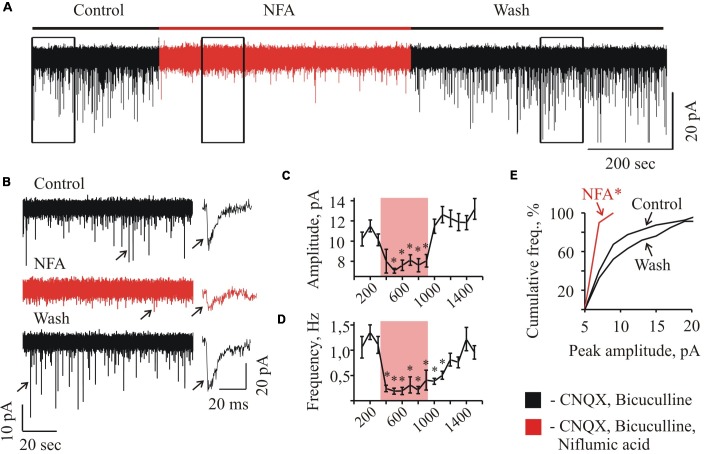
Effect of NFA on sIPSCs recorded from “neonatal” HMs. **(A)** Trace of continuous whole-cell recording of sIPSCs, illustrating the effect of 100 μM NFA at HM from P3 mice. The duration of NFA application is highlighted by red. V_hold_ = –70 mV. **(B)** Examples of segments from the trace **(A)** indicated by rectangular frames at 10-fold faster time scale; recording was performed in the control conditions (a), in the presence of NFA (b), and during wash (c). On the right single events are shown (marked on traces by arrows) at1000-fold higher time resolution. Graphs showing the time course of the development of NFA effect on the amplitude **(C)** and frequency **(D)** of spontaneous glycinergic IPSCs. Each point represents the mean ± SEM of values during 100 s. Duration of NFA action is highlighted by transparent red. ^∗^ Significant difference from “Control” with *P* < 0.05 (one-way ANOVA test with Bonferroni correction). **(E)** Cumulative frequency distribution of peaks amplitudes. Note drastically decreased number of sIPSCs with amplitude more than 10 pA in the presence of NFA.^∗^ Significant difference from “Control” and “Wash” with *P* < 0.05 (two sample Kolmogorov–Smirnov test).

**FIGURE 3:**
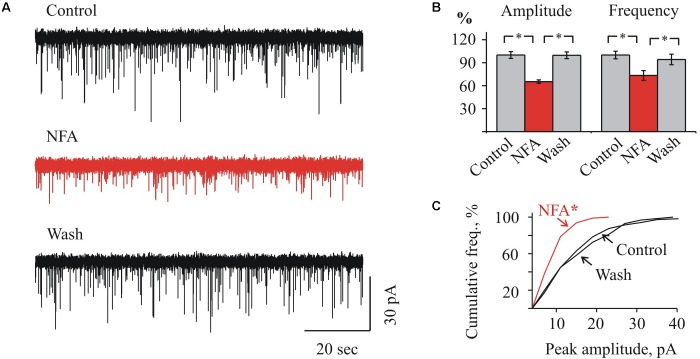
Effect of NFA on sIPSCs in “juvenile” HMs. **(A)** Representative traces of sIPSCs whole-cell recordings in control (top), 1.7 min after addition of 100 μM NFA (middle) and 10 min of wash with aCSF (bottom). P9 HM. V_hold_ = –70 mV. **(B)** Mean percent ± SEM of sIPSCs amplitude (left) and frequency (right) inhibition by 100 μM NFA. ^∗^ Significant difference with *P* < 0.05 (one-way ANOVA test with Bonferroni correction). **(C)** Cumulative frequency distribution of peaks amplitudes in control (a) during NFA application (b) and wash (c). ^∗^ Significant difference from “Control” and “Wash” with *P* < 0.05 (two sample Kolmogorov–Smirnov test).

To obtain the pure glycinergic synaptic events, CNQX and bicuculline were added systematically to the aCSF. In these conditions, stable IPSCs (range in different cells 5–200 pA among spontaneous IPSCs and up to 600 pA among eIPSCs) were recorded at -70 mV or +30 mV (Figures 1C,D,H, 2A,B, 3A,B, 5A,B), and their activity was completely abolished by 2 μM strychnine, an antagonist of GlyRs (Figures 1H, 5A).

Inhibitory post-synaptic current deactivation kinetics was best fit with a single exponential function. The decay time constant varied in the different experimental conditions from 6 to 20 ms, consistently with the previous observations (Singer et al., 1998; Mukhtarov et al., 2005). As it was mentioned already, the previous studies demonstrated that the early postnatal development is accompanied by the changes in subunit composition of the chloride (Cl^-^)-permeable GlyR channels, resulting in a decrease of “slow fetal” alpha2 and an elevation of “fast adult” alpha1 subunits (Singer et al., 1998). Consequently, a kinetics of glycinergic currents became faster during the first two postnatal weeks. We confirmed these observations analyzing a decay time constant (τ_decay_) of the induced glycinergic IPSCs in the two age mice groups. In the “neonatal” group, a τ_decay_ was significantly longer comparing to the “juvenile” one: 10.8 ± 0.6 ms (*n* = 8) and 6.3 ± 0.4 ms (*n* = 10), respectively (Figure 1E). In addition, with aging the amplitude and frequency of sIPSCs increased (Figures 1F,G). The mean amplitude has grown from 10,2 ± 0,7 to 13,1 ± 1,1 pA and the mean frequency elevated from 0,85 ± 0,21 to 2,1 ± 0,2 Hz in the “neonatal” and the “juvenile” groups, respectively (*n* = 6).

These observations provided us the background for the analysis of the NFA effects on the glycinergic IPSCs at the early postnatal development.

### Action of NFA on the Amplitude and Frequency of Spontaneous Glycinergic IPSCs

At long-lasting recordings from the neonate (P2–P4) MNs, application of 100 μM NFA caused a reversible decrease in the amplitude and frequency of spontaneous glycinergic IPSCs (Figure 2). In different cells, the maximum effect of inhibition was achieved within 1–3 min after addition of NFA. Traces illustrating these changes in the glycinergic activity at a faster time scale are shown in Figure 2B. Obviously, in the presence of NFA the spontaneous activity is strongly suppressed (trace b).

Figure 2 illustrates the effect of NFA on the MN in neonatal slice (P3) at V_hold_ = -70 mV. Addition of NFA caused decrease in the mean amplitude of sIPSCs from 10.2 ± 0.7 to 7.1 ± 0.2 pA (to 69.6 + 2.0% see the graph C) and the frequency reduction from 1.1 ± 0.2 to 0.2 ± 0.1 Hz (to 17.1 ± 5.3% see the graph D) (Figures 2C,D). In the different cells, the maximum effect of inhibition was achieved within 1–3 min after addition of NFA and the complete recovery was observed following 6–12 min of the washout (Figures 2C,D). Cumulative interevent interval plot of the glycinergic sIPSCs amplitudes (Figure 2E) shows a strong decrease in the frequency of high amplitude events. NFA completely abolished the events above 10 pA, while in the control conditions sIPSCs up to 20 pA were frequently observed.

In MNs from the juvenile brainstem slices, NFA also caused a suppression of sIPSCs activity, however, the effect was less pronounced. As Figures 3A,B illustrate, in MNs from P9 mice application of NFA resulted in a reduction of the average IPSCs amplitude from 16.2 ± 0.7 to 10.6 ± 0.4 pA (to 65.3 ± 2.2%) and the frequency decrease from 2.2 ± 0.1 to 1.6 ± 0.1 Hz (to 73.3 ± 6.4%). This illustrates also the cumulative interevent interval plot of the glycinergic sIPSCs amplitudes (Figure 3C).

**FIGURE 4:**
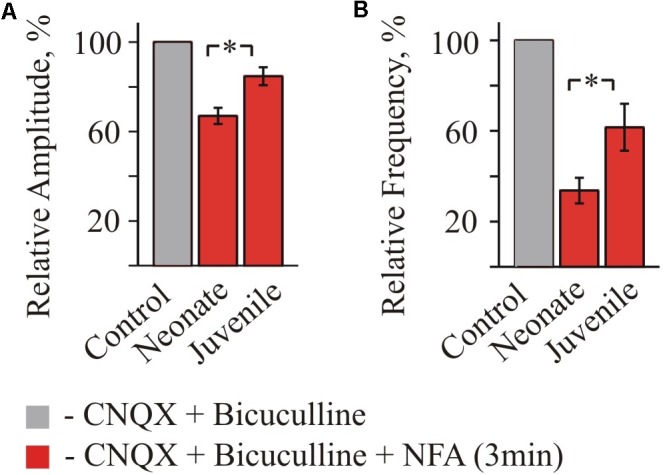
Summary of inhibitory effect of NFA on spontaneous glycinergic IPSCs in HM from “neonatal” and “juvenile” age groups. Average percentage of sIPSCs amplitude **(A)** and frequency **(B)** decrease during application of 100 μM NFA. Recordings in the presence of 20 μM bicuculline and 10 μM CNQX at V_hold_ = –70 mV. Data from nine “neonatal” and from seven “juvenile” MNs. ^∗^ Significant difference from “Control” (*P* < 0.05). One-way ANOVA test with Bonferroni correction.

Summary analysis from 3 to 4 neurons of the both age groups (Figure 4) has shown that for the neonate MNs, the mean amplitude of IPSCs after 2 min of incubation in NFA decreased to 62.9 ± 3.4% (*P* < 0.05) and the mean frequency to 22.02 ± 4.77% (*P* < 0.05) (*n* = 3). For the juvenile group, incubation in NFA caused a decrease of the mean amplitude and frequency to 76.8 ± 6.8 and 64.4 ± 18.6%, respectively (*n* = 4).

Altogether, these data demonstrate that NFA causes a suppression of the amplitude and frequency of spontaneous glycinergic IPSCs and its inhibitory potential decreases on the second week of postnatal development.

### Effect of NFA on Evoked Glycinergic IPSCs

At a heterologous expression of GlyRs, NFA inhibits the glycine-induced currents in a voltage-dependent manner, with a higher blocking potency at the positive membrane potentials (Maleeva et al., 2017). It has been also shown that NFA can block some types of the voltage-gated Ca^2+^ channels of T-type (Cav3) (Balderas et al., 2012). As neurotransmitter release is highly controlled by influx of Ca^2+^ in the presynaptic terminals, decreasing of the spontaneous glycinergic IPSCs might result, at least partially, from the inhibition of presynaptic voltage-gated Ca^2+^ channels.

To clarify this question and also to analyze the voltage-dependence of NFA action on the glycinergic IPSCs in the brain slices of different ages, we recorded the stimulation induced glycinergic IPSCs under the different holding potentials: V_hold_ = + 30 or -70 mV. For this purpose we used a pair-pulse stimulation, delivering two consecutive pulses separated by 50 ms to the stimulating electrode.

**FIGURE 5:**
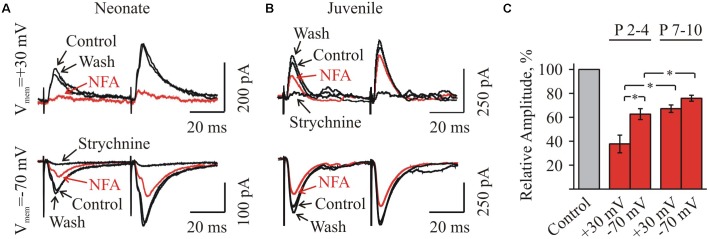
Effect of NFA on evoked glycinergic IPSCs in neonatal and juvenile HMs at different membrane potentials. **(A,B)** Examples of eIPSCs at pair-pulse stimulation in control condition, under 100 μM NFA action and after washing (as indicated by arrows) in the neonatal **(A)** and juvenile **(B)** MNs. Whole-cell recordings at holding potentials +30 mV (top traces) and –70 mV (bottom traces). Recording made after 3 min of incubation in NFA are shown in red. For each age traces at positive and negative potentials are shown from the same motoneuron. Notice stronger inhibition for neonatal motoneuron currents at positive potential and weaker effect of NFA on juvenile cell. To ensure that eIPSCs were glycinergic, at the end of each experiment eIPSCs were induced in the presence of strychnine. **(C)** Summary of the percentage of decrease of the amplitudes of eIPSCs under NFA application in the neonatal (*n* = 4) and juvenile (*n* = 5) groups, at two different holding potentials: –70 mV and +30 mV. Values are presented as percentages from initial levels. ^∗^Significant difference (*P* < 0.05).

Firstly, we analyzed the voltage-dependence of NFA action on the amplitude of glycinergic eIPSCs. In the group P3–P4, the mean amplitude of glycinergic eIPSCs by 3 min of the incubation in 100 μM NFA decreased to 37.8 ± 7.4 and 62.6 ± 4.8% (*n* = 4) at V_hold_ = + 30 and -70 mV, respectively (Figures 5A,C). In the group P7–P10, the inhibition was less pronounced, while still voltage dependent. The mean amplitude of glycinergic eIPSCs in the presence of 100 μM NFA decreased to 67.2 ± 3.1 and 75.9 ± 2.5% (*n* = 5), at +30 and -70 mV, respectively (Figures 5B,C).

Next, we analyzed the effect of NFA on the relative amplitudes of IPSCs induced by a pair-pulse stimulation protocol at V_hold_ -70 mV. In the majority of neurones, we observed an increment in the amplitude of IPSC evoked by the second stimulus with respect to the first one. The average value of a pair-pulse ratio, expressed as the ratio of IPSC amplitudes I_2_/I_1_, in the neonate and juvenile groups was 179.7 ± 8.5% (*n* = 4) and 131.5 ± 17.3% (*n* = 7), respectively (data not shown), indicating a pair-pulse facilitation.

Accordingly to the residual calcium hypothesis (Katz and Miledi, 1968), a pair-pulse facilitation results from the presence of residual Ca^2+^ in the presynaptic terminals, which remains for several 100 ms after the first stimulus and adds significantly to the Ca^2+^ rise in the terminal, increasing the probability of the neurotransmitter release during the repeated stimulation (Manabe et al., 1993; Son and Carpenter, 1996).

**FIGURE 6:**
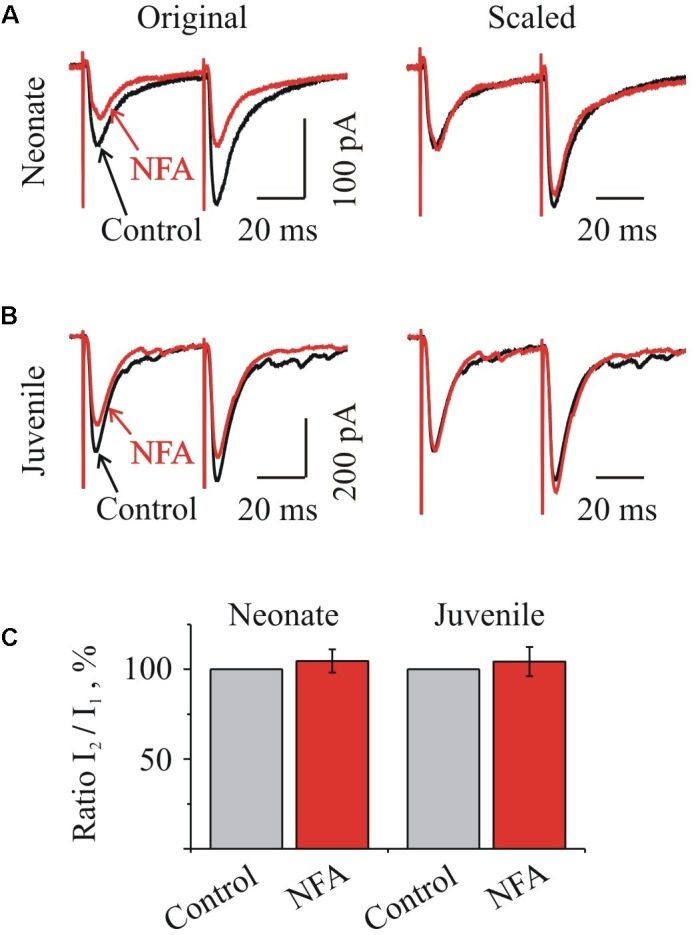
Niflumic acid did not change the ratio of IPSCs at pair-pulse stimulation. **(A,B)** Traces of glycinergic IPSCs evoked by a paired stimulation (50 ms interval) before (Control) and during NFA application (NFA) to “neonatal” **(A)** and “juvenile” brainstem slices. *Left*: examples of superimposed traces, *Right:* same traces, scaled to control amplitude of the first stimulation. Notice absence of changes in the amplitude of the second pulse during the action of NFA. Recording from P3 and P10 HMs. **(C)** Summary of relative I_2_/I_1_ ratio during NFA action in percentages from initial levels for the “neonatal” (*n* = 4) and “juvenile” (*n* = 7) MNs, as indicated. Mean ± SEM.

Concerning our experiments, if NFA modulates an activity of presynaptic voltage-gated Ca^2+^ channels, one would expect a decrease of the residual calcium concentration and consequent changes in a pair-pulse ratio. However, as Figure 6 illustrates, NFA had no effect on the I_2_/I_1_ ratio either in the neonate (Figures 6A,C) or juvenile (Figures 6B,C) groups of MNs.

The average relative values of a I_2_/I_1_ ratio in the neonate and juvenile groups in the presence of 100 μM NFA increased only by 4.6 ± 6.4% (*n* = 4) and 4.4 ± 8.1% (*n* = 7) respectively (Figure 6C).

These results suggest that NFA does not modulate the function of the presynaptic voltage-gated Ca^2+^-channels and the probability of the neurotransmitter release from the presynaptic terminals, supporting the post-synaptic origin of the glycinergic IPSCs inhibition by this compound.

## Discussion

In this study, we analyze the action of NFA on the glycinergic synaptic currents in the HMs from brain slices of mice of the different postnatal age groups. Recording from the HMs of brainstem slices of the “neonatal” and the “juvenile” mice under the conditions of constant suppression of the glutamatergic and GABAergic neurotransmission, we have demonstrated that: (i) NFA caused an inhibition of the amplitude and frequency of the spontaneous synaptic glycinergic currents and the amplitude of eIPSCs; (ii) inhibitory ability of NFA was weaker in the “juvenile” group; (iii) inhibition was voltage-dependent with the higher efficacy at the positive potentials and this effect was particularly pronounced on the MNs from the “neonatal” group; (iv) NFA did not change the degree of a paired-pulse potentiation of eIPSCs in the both age groups.

Recent study at a heterologous expression of the different homomeric and heteromeric GlyRs demonstrated that NFA is an inhibitor of GlyRs with a subunit specificity of action, being relatively weak on GlyRs formed by alpha1 subunits (Maleeva et al., 2017). The effect of NFA was voltage-dependent with a higher affinity to the receptor at the positive potentials, which was particularly pronounced at alpha2 GlyRs. The present observations, on the decrease of NFA ability to inhibit the glycinergic IPSCs in the “juvenile” group demonstrate that in the brain slices also NFA has lower affinity to alpha1 GlyRs comparing to alpha2.

Our results are consistent with the early studies on the predominant presence of alpha2 GlyRs at birth and the decrease of their number during the first weeks of postnatal life, contrary to alpha1 GlyR subunits, which expression level is low at birth and rapidly increases with the age (Becker et al., 1988; Malosio et al., 1991; Watanabe and Akagi, 1995; Singer et al., 1998). This was also supported by our analysis of the voltage-dependence of the NFA action on the glycinergic synaptic currents.

The voltage-dependent inhibitory activity of NFA with a lower blocking efficiency at the negative membrane potentials is known since the study describing an interaction of NFA with the calcium-activated chloride channels (Hogg et al., 1994). Recent patch-clamp study of GlyRs of the known subunit composition demonstrated that a voltage-dependence of NFA action on the homomeric and heteromeric alpha2 GlyRs is much higher than the one of alpha1 GlyRs (Maleeva et al., 2017). Recording the glycineregic eIPSCs from the HMs of brainstem slices we found that at changing membrane potential by 100 mV (from +30 to -70 mV), the blocking effect of NFA decreased by 1,7- and 1,3-fold for the “neonate” and the “juvenile” groups, respectively. This suggests the substantial expression of alpha1 subunits already in the first days of the postnatal life and it predominance in the “juvenile” HMs.

Inhibitory effect of NFA, at least partially, could originate from the modulation of the presynaptic voltage-gated Ca^2+^ channels. It is known that a short-term facilitation is caused by the residual Ca^2+^ that builds up from the action potentials generated by a previous pulse (Katz and Miledi, 1968; Zucker and Regehr, 2002) and Ca^2+^ entry through the voltage-gated Cav2 channels is required for a synaptic facilitation (Son and Carpenter, 1996; Inchauspe et al., 2007). Recently it has been shown that NFA causes an inhibition of the voltage-gated Ca^2+^ T-type channels in the voltage-independent manner (Balderas et al., 2012). In the new nomenclature of the voltage-gated Ca^2+^ channels (Ertel et al., 2000) T-type channels belongs to the Ca_V_3 subfamily and have been identified in several tissues, including neural tissue, heart, kidney, smooth muscle, skeletal muscle, sperm, and also in some specific types of neurons (Perez-Reyes, 2003; Darszon et al., 2006). Historically, voltage-gated Ca^2+^ channels were divided in the two major classes: the LVA and the HVA channels (Llinas and Yarom, 1981; Nowycky et al., 1985). LVA or T-type channels belongs to the subfamily of Ca_V_3 channels; HVA are classified into L-, N-, P/Q-, and R types depending on their biophysical properties (Perez-Reyes, 2003) and the major source of Ca^2+^ entry for initiating of the synaptic transmission, represent HVA channels of Ca_V_2 subfamily (Catterall et al., 2013).

To check whether NFA, similarly to Ca_V_3, could modulate a function of Ca_V_2 subfamily channels, causing the changes of the neurotransmitter release probability, we analyzed its action on a paired-pulse facilitation of the glycinergic currents. At application of the two consecutive pulses separated by 50 ms, the amplitude of IPSC amplitude evoked by the second stimulus was usually higher, indicating a short-term facilitation. In our experiments NFA caused an inhibition of both eIPSCs with a similar degree of an amplitude decrease in all studied MNs.

Thus, our results suggest that NFA does not modulate a function of the presynaptic voltage-gated Ca^2+^-channels, acting primarily on the post-synaptic GlyRs in the voltage-dependent manner, i.e., its inhibition of glycinergic IPSCs in HMs has a purely post-synaptic nature.

## Author Contributions

EP, DP, GM, and PB performed the experiments, analyzed data, and wrote the manuscript. MM reviewed and discussed the manuscript.

## Conflict of Interest Statement

The authors declare that the research was conducted in the absence of any commercial or financial relationships that could be construed as a potential conflict of interest.

## Abbreviations

aCSF: artificial cerebrospinal fluid
CNQX: 6-cyano-7-nitroquinoxaline-2,3-dione
CNS: central nervous system
eIPSCs: evoked inhibitory post-synaptic currents
GlyRs: glycine receptors
HM: hypoglossal motoneurones
HVA: high voltage-activated
IPSCs: inhibitory post-synaptic currents
LVA: low voltage-activated
NFA: niflumic acid
sIPSCs: spontaneous inhibitory post-synaptic currents
V_hold_: holding membrane potential
